# A new synonym in the subfamily Thrigmopoeinae Pocock, 1900 (Araneae, Theraphosidae)

**DOI:** 10.3897/zookeys.749.23414

**Published:** 2018-04-10

**Authors:** Pradeep M. Sankaran, Pothalil A. Sebastian

**Affiliations:** 1 Division of Arachnology, Department of Zoology, Sacred Heart College, Thevara, Cochin, Kerala 682 013, India

**Keywords:** India, junior synonym, polychromatism, taxonomy, Western Ghats

## Abstract

As the species *Haploclastus devamatha* Prasanth & Jose, 2014 is indistinguishable from *Thrigmopoeus psychedelicus* Sanap & Mirza, 2014, the latter is herein considered junior synonym of the former. Occurrence of polychromatism in *H. devamatha* is noted, and two distinct colour morphs of the species are recognised, a pink form and a blue form. The natural history and conservation of the species are discussed and its known distribution is updated.

## Introduction

The subfamily Thrigmopoeinae Pocock, 1900, a group of large, ground-dwelling, burrowing mygalomorph spiders endemic to the Western Ghats of India (Mirza and Sanap 2013), is the smallest of all the eight theraphosid subfamilies (Raven 1985) and currently comprises ten nominal species under two genera: *Haploclastus* Simon, 1892 (with seven species) and *Thrigmopoeus* Pocock, 1899 (with three species) (World Spider Catalog 2018). Though the genus *Haploclastus* is numerically rich, all the described species except *Haploclastus tenebrosus* Gravely, 1935 and *Haploclastus validus* (Pocock, 1899), are known only from original descriptions and most of them lack detailed descriptions and illustrations (Simon 1892, Pocock 1899, Gravely 1915, Barman 1978). Recent taxonomic treatment of *Haploclastus* (Siliwal and Raven 2010) indicates the possibility of uncertain placement of species within the subfamily. Similarly, *Thrigmopoeus* species are difficult to distinguish from *Haploclastus* species with morphological features. In the present paper, a proposal to synonymise *Thrigmopoeus psychedelicus* Sanap & Mirza, 2014 with *Haploclastus devamatha* Prasanth & Jose, 2014 is presented. Additionally, the current distribution of *H. devamatha* is mapped.

## Materials and methods

The specimens were studied under a Zeiss Stemi 2000-C stereomicroscope. Drawings were made by the aid of a drawing tube attached to the microscope. Field photos were taken with Canon EOS 6D camera with Canon 100mm Macro photo lens. The specimens are deposited in a reference collection housed at the Division of Arachnology, Department of Zoology, Sacred Heart College, Thevara, Cochin, Kerala, India (**ADSH**).

## Taxonomy

### Theraphosidae Thorell, 1869

#### Thrigmopoeinae Pocock, 1900

##### *Haploclastus* Simon, 1892

###### Haploclastus devamatha

Taxon classificationAnimaliaAraneaeTheraphosidae

Prasanth & Jose, 2014

**Figure 1.**
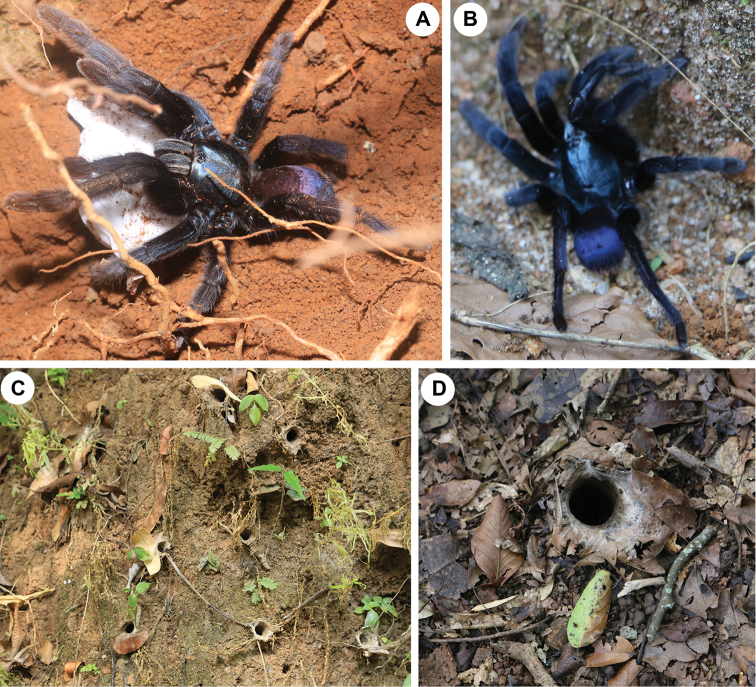
*Haploclastus devamatha* Prasanth & Jose, 2014 **A** female with egg sac from Thenmala (*pink form*), dorso-retrolateral **B** female from Thenmala (*blue form*), dorsal **C** active burrows of juveniles on the road side mud embankment, Thenmala **D** active burrow of adult female on the forest floor, Kulathupuzha Forest Reserve. Photo credit Jimmy Paul.

**Figure 2.**
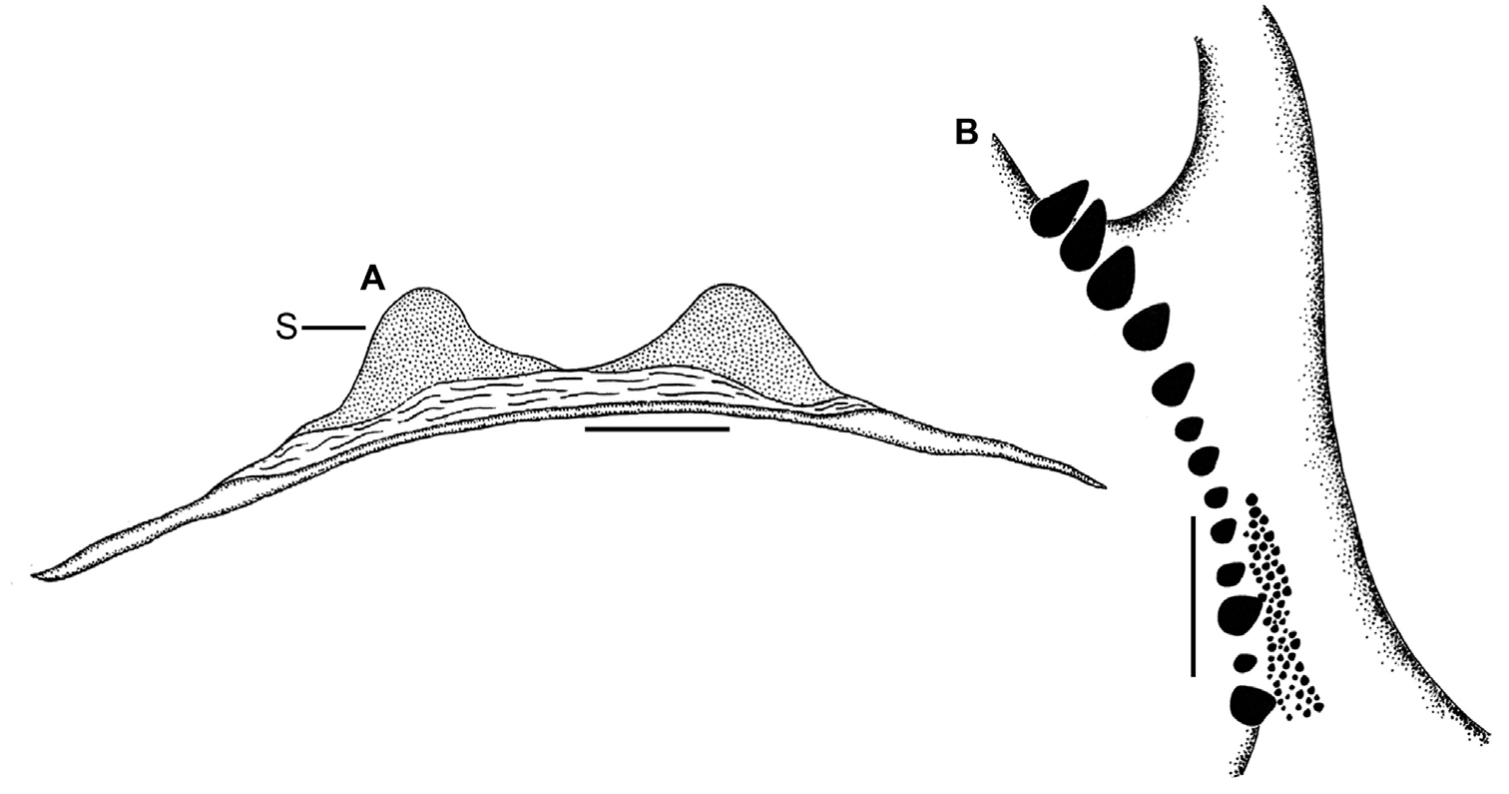
*Haploclastus devamatha* Prasanth & Jose, 2014 **A** genitalia, dorsal **B** left chelicera, prolateral showing teeth arrangement. **S** = Spermatheca. Scale bars: 1 mm (**A**); 2 mm (**B**).

**Figure 3.**
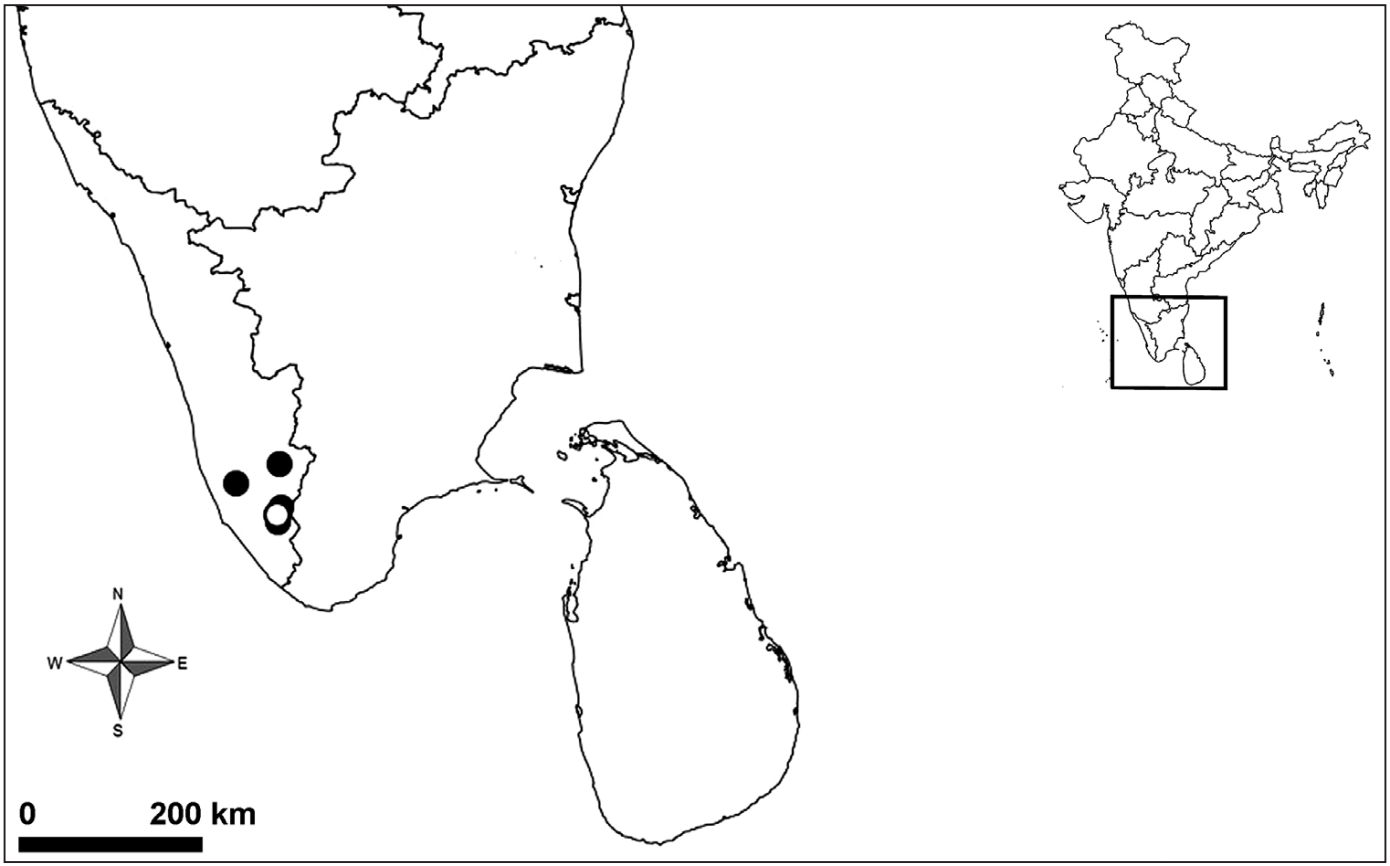
Current distribution of *Haploclastus devamatha* Prasanth & Jose, 2014 ○ new record, ● literature records (Prasanth and Jose 2014, Sanap and Mirza 2014).

Figs 1A–D , 2A–B , 3

Haploclastus devamatha Prasanth & Jose, 2014: 495, figs 1, 2A–I, 3A–D, 4A–D (Description and illustration of female).
Thrigmopoeus psychedelicus Sanap & Mirza, 2014: 481, figs 1, 2a–d, 3a–c, 3e, 4 (Misidentification; description and illustration of female). **New synonym**.

####### Type material.

Holotype female of *H. devamatha* (DMCK 13/110) from INDIA: *Kerala*: Kollam: Kulathupuzha Forest Reserve, 8°54'6.37"N, 77°3'51.70"E, 134 m alt., Prasanth M. T. & Sunil Jose K. leg., 31 July 2013, repository Deva Matha College, Kuravilangad, Kerala (DMCK), not examined. Paratype female collected together with the holotype deposited in the reference collection of Sacred Heart College, Thevara (ADSH101501), examined.

Holotype female of *T. psychedelicus* (BNHS SP115) from INDIA: *Kerala*: Kollam: near Thenmala: Ambanad Tea Estate, 9°2'18"N, 77°5'22"E, 561 m alt., Rajesh Sanap, Zeeshan Mirza & Karthik Prabhu leg., 22 December 2013, repository Bombay Natural History Society, Mumbai, (BNHS), not examined.

####### Other material examined.

**INDIA**, Kerala: Kollam: Thenmala, 8°57'30.7"N, 77°10'38.9"E, 567 m alt., 10 January 2015, M. S. Pradeep leg., from burrows on mud embankment, by hand: 2 females (ADSH101502) (NEW RECORD); Kulathupuzha Forest Reserve, 8°54'6.37"N, 77°3'51.70"E, 134 m alt., 11 January 2015, M. S. Pradeep leg., from burrows on mud embankment and forest floor, by hand: 4 females, 3 subadult females (ADSH101503).

####### Description.

For description and other details of the species, see Sanap and Mirza (2014).

####### Justification of the synonymy.

Although the types of *T. psychedelicus* were not examined, good illustrations and images of this species are available (Sanap and Mirza 2014: figs 1, 2a–d, 3a–e). In the original description of *H. devamatha*, Prasanth and Jose (2014) pointed out several diagnostic somatic features for this species. The first and most important diagnostic character refers to the body colouration of this species, which has iridescent blue and pink colouration. Sanap and Mirza (2014) also noted the same body colouration for *T. psychedelicus* (compare Prasanth and Jose 2014: fig. 1 with Sanap and Mirza 2014: fig. 4). The original illustrations of cheliceral and maxillary lyrae of *T. psychedelicus* are exact matches with the colour photographs of the same provided for *H. devamatha* by Prasanth and Jose (2014) (compare Sanap and Mirza 2014: fig. 3a–c with Prasanth and Jose 2014: figs 3B, 3D, 4A–B). Though the spermathecae of *H. devamatha* (Prasanth and Jose 2014: fig. 2F) seem quite different from that of *T. psychedelicus*, detailed examination of the paratype and topotypes of *H. devamatha* reveals that their illustration is imperfect and misleading, and that both these specimens indeed belong to the same species. The species *T. psychedelicus* should thus be regarded as a junior synonym of *H. devamatha*.

####### Note.

Prasanth and Jose published their findings in January 2014, whereas Sanap and Mirza published their discovery in July 2014, so priority must go to the name *Haploclastus devamatha* and the name *Thrigmopoeus psychedelicus* becomes its junior synonym.

####### Distribution.

India (Kerala: Kollam, Pathanamthitta) (Fig. 3).

####### Polychromatism.

Females of *H. devamatha* are remarkable for their polychromatism (Sanap and Mirza 2014). Two distinct colour forms have been observed in the population of *H. devamatha*: a ‘pink form’ with bluish prosoma and pinkish opisthosoma and the ‘blue form’ with uniform bluish black prosoma and opisthosoma (Fig. 1A–B). Perhaps this change in colour is related to the age of the spider as suggested by Sanap and Mirza (2014), but confirmation requires further investigations.

####### Natural history and conservation.

*Haploclastus devamatha* builds unbranched burrows lined with silk. The burrows have single entrance, which is a circular opening ornamented with dried leaves pasted together using silk to form a short turret (Fig. 1D). As noted by Sanap and Mirza (2014), the burrows are found to occur on the roadside mud embankments inside and nearby regions of the forests at a height of 1–6 metres from the ground (Fig. 1C). Rarely, adult burrows are observed on the forest floors. In the Thenmala and Kulathupuzha regions, we were able to locate a large number of juvenile and subadult burrows that are built on the roadside mud embankments. Within a stretch of 2 kilometres in the Thenmala region, 110 burrows were observed and at a stretch of 1.5 kilometres in the Kulathupuzha region, 52 burrows were found. The tendency of this species to build its burrows predominantly on the roadside mud embankments points to the fact that its survival is under threat due to the common anthropogenic activities like soil removal from the mud embankments and burning dried leaves gathered together near the mud embankments.

## Supplementary Material

XML Treatment for Haploclastus devamatha
